# A note on the stability of surfaces along null cones under area-preserving variations

**DOI:** 10.1007/s11005-026-02184-3

**Published:** 2026-11-11

**Authors:** Markus Wolff

**Affiliations:** Faculty of Mathematics, University of Vienna, Oskar-Morgenstern Platz 1, Vienna, 1090 Vienna Austria

**Keywords:** Prescribed curvature, Stability, Null Geometry, 53C42, 53C21, 53C24

## Abstract

In this note, we investigate a notion of stability for spacelike cross sections of a null cone under area-preserving variations that has been introduced in previous work by Kröncke and the author. Here, we consider null cones, i.e., null hypersurfaces with non-vanishing expansion, with spherical cross sections in a 4-dimensional spacetime and show that the Hawking energy of a stable cross section admits a nonnegative lower bound provided the dominant energy condition holds. Similar to a recent work by Peñuela Diaz, we show that under an additional assumption the Hawking energy is zero if and only if the stable cross section embeds isometrically into the Minkowski lightcone. As a main result, we show that the only stable cross sections of the standard Minkowski lightcone are round spheres.

## Introduction

In Riemannian geometry, many fundamental notions of stability arise directly from a geometric variational problem. While the perspective from an underlying variational problem often breaks down when considered in a more general setup, analogous notions of stability have been studied with great success in the context of general relativity. In particular, various notions of stability have been considered in close analogy to the stability for minimal and constant mean curvature surfaces in Riemannian geometry. See, for example, [1–9] for a non-exhaustive list.

Given a spacelike codimension-2 surface Σ in an ambient spacetime $(\overline{M},\overline{g})$, possibly restricted to a given initial data set (*M*, *g*, *K*) or a given null hypersurface $\mathcal {N}$, one can consider the *null expansions* $\underline{\theta }$, θ with respect to a null frame $\{\underline{L},L\}$ of $\Gamma (T^\perp \Sigma )$ and the *spacetime mean curvature* $\mathcal {H}^2$, given as the Lorentzian length of the codimension-2 mean curvature vector, as suitable generalizations to the concept of mean curvature for a hypersurface in a Riemannian manifold. In particular, marginally trapped surfaces, where either $\underline{\theta }$ or θ vanish identically, present themselves as the natural analogue to minimal surfaces. The stability of marginally outer trapped surfaces (MOTS) and generalizations of MOTS stability have been, for example, studied in [1–8] in analogy to the stability of minimal surfaces.

Here, we study a notion of stability under area-preserving variations for spacelike cross sections Σ of a given null hypersurface $\mathcal {N}$. More precisely, we restrict our attention to null cones [10], i.e., the expansion of $\mathcal {N}$ is strictly positive with respect to a choice of null generator $\underline{L}$. We note that this directly implies that $\underline{\theta }>0$ for every spacelike cross section Σ with respect to $\underline{L}$. In view of the typical toy models, such as the Minkowski and Schwarzschild lightcone, it is a natural assumption to make when not considering non-expanding horizons [10], i.e., models for black hole and cosmological horizons. We note, however, that in general a null hypersurface is neither.

We say a spacelike cross section Σ of a null cone $\mathcal {N}$ is stable (under area-preserving variations) if

$$ \int _\Sigma f J(f)\,\textrm{d}\mu \ge 0 $$

for all $f\in W^{1,2}(\Sigma )$ such that $\int _\Sigma f\,\textrm{d}\mu =0$, where the *Jacobi operator* *J* is derived from the linearization of the spacetime mean curvature $\mathcal {H}^2$. See [11]. We note that *J* is gauge-invariant and therefore purely determined by the geometry of Σ. While the condition $\int _\Sigma f\,\textrm{d}\mu =0$ is commonly associated with volume-preserving variations in Riemannian geometry, in particular in the context of CMC stability, there is a natural relation to area-preserving variations on null cones. In fact, we note that there is likely no well-defined notion of enclosed volume in the null case. In this sense, although not arising from an underlying variational problem, the above notion of stability presents itself as a natural null analogue to CMC stability in the Riemannian setting. See Sect. 3 for details. We note further that the leaves of the asymptotic foliation by surfaces of constant spacetime mean curvature (STCMC) of an asymptotically Schwarzschildean lightcone constructed in [11] are strictly stable in the above sense.

Here, we also mention the work of Alaee, Lesourd and Yau [1] and work of Peñuela Diaz [9] who consider similar notions of stability for codimension-2 surfaces in an ambient spacetime. In fact, the notion of ${\textbf {H}}$-stability (under a volume-preserving condition) in [1] is equivalent to the notion of stability considered here if taken with respect to the null direction $\underline{\theta }^{-1}\underline{L}$.

As a first result, we show that a stable spacelike cross section Σ with $\mathcal {H}^2\ge 0$ of a null cone $\mathcal {N}$ satisfies

$$ \int _\Sigma \mathcal {H}^2\,\textrm{d}\mu \le 16\pi $$

if the ambient spacetime satisfies the dominant energy condition. In particular,

$$ m_H(\Sigma ):=\sqrt{\frac{\left| \Sigma \right| }{16\pi }}\left( 1-\frac{1}{16\pi }\int _\Sigma \mathcal {H}^2\,\textrm{d}\mu \right) \ge 0, $$

where $m_H(\Sigma )$ is the *Hawking energy* of Σ. See Proposition 1. This can be understood as the null analogue to an estimate by Christodoulou and Yau [12] for stable CMC surfaces. Note that due to the equivalence to ${\textbf {H}}$-stability in a suitable null direction, this also directly follows from [1, Theorem 3.3]. See also [9, Theorem 4.1] for a related result. We also provide a rigidity statement under an additional assumption in a similar spirit to [9, Theorem 4.1], see Proposition 2.

As a main result (Corollary 4), we show that any stable spacelike cross section of the standard Minkowski lightcone is an STCMC surface, i.e., a round sphere with constant curvature. This can be understood as the analogue to the well-known fact that any stable topological 2-sphere with constant mean curvature in $\mathbb {R}^3$ is a round sphere. The proof relies on a new set of test functions that are derived from a Minkowskian 4-vector for spacelike cross sections introduced by the author in [13].

Further motivated by the geometry of the Minkowski spacetime, we consider a family of area-preserving variations defined via Lorentz transformations. While we do not give an immediate application in the context of stability, we obtain an integral identity (Proposition 5) as a direct consequence of the Kazdan–Warner identity.

This paper is structured as follows: In Sect. 2, we briefly summarize some necessary prerequisites. In Sect. 3, we introduce the Jacobi operator *J* and define stability on a null cone. We further show that the Hawking mass is nonnegative for stable spacelike cross sections provided the dominant energy condition holds and provide a rigidity statement under an additional assumption. In Sect. 4, we look at the stability operator in a class of highly symmetric spacetimes and prove our main result. Finally, we consider another family of area-preserving variations motivated by Lorentz transformations in Sect. 5. In addition to deriving an integral identity for metrics on $\mathbb {S}^2$, we collect all the necessary identities for applications in the future.

## Preliminaries

In the following, $(\overline{M},\overline{g})$ will always denote a 4-dimensional oriented spacetime, i.e., a time-oriented and oriented Lorentzian manifold with signature (-,+,+,+). In particular, we always assume that $(\overline{M},\overline{g})$ admits a globally defined, timelike vector field $X\in \Gamma (TM)$ which induces the well-known notions of future-directed, respectively, past-directed vectors, vector fields, and curves. We recall that $(\overline{M},\overline{g})$ is said to satisfy the *dominant energy condition* (DEC) if

G(Y,Z)≥0

for all future-directed causal (timelike or null) vector fields $Y,Z\in \Gamma (T\overline{M})$, where the Einstein tensor *G* is given by

$$ {G}(Y,Z):=\overline{\textrm{Ric}}(Y,Z)-\frac{1}{2}\overline{\textrm{R}}\,\overline{g}(Y,Z). $$

Note that the dominant energy condition directly implies the null energy condition (NEC), i.e.,

$$ \overline{\textrm{Ric}}(L,L)\ge 0 $$

for all null vector fields $L\in \Gamma (T\overline{M})$. Here, we use the following conventions for the Riemann curvature tensor $\operatorname {Rm}$, Ricci curvature $\textrm{Ric}$, and scalar curvature $\textrm{R}$ of a semi-Riemannian manifold (*M*, *g*):

$$\begin{aligned} \operatorname {Rm}(X,Y,W,Z)&=g(\nabla _X\nabla _YZ-\nabla _Y\nabla _XZ-\nabla _{[X,Y]}Z,W),\\ \textrm{Ric}(X,Y)&=\text {tr}_g\operatorname {Rm}(X,\cdot ,Y,\cdot ),\\ R&=\text {tr}_g \textrm{Ric}. \end{aligned}$$

We say an oriented hypersurface $\mathcal {N}$ in $(\overline{M},\overline{g})$ is a null hypersurface if its induced metric is degenerate. Equivalently, there exists a non-vanishing vector field $\underline{L}\in \Gamma (T\mathcal {N})$ such that $\underline{L}$ is orthogonal to all tangent directions, i.e.,

$$ T_p\mathcal {N}=(\underline{L}_p)^\perp \subseteq T_p\overline{M}\text { for all }p\in \mathcal {N}. $$

In particular, $\underline{L}$ is orthogonal to itself and therefore null. In the following, we briefly recall some well-known properties of null hypersurfaces relevant to our discussion without proof. For a more thorough introduction into null hypersurfaces using the same notation and conventions, we refer the interested reader to [14, Section 4.7]. See also the references given therein, e.g., [15–17]. First, we note that $\mathcal {N}$ is ruled by the integral curves of $\underline{L}$ and call $\underline{L}$ a null generator. The choice of null generator is not unique^1^ and $\underline{L}$ can (at least locally) always be chosen such that its integral curves are null geodesics.^2^ Hence, a null hypersurface $\mathcal {N}$ is ruled by null geodesics. The null second fundamental form of $\mathcal {N}$ (with respect to $\underline{L})$ is defined as

$$ \underline{\chi }(X,Y):=-\overline{g}(\overline{\nabla }_XY,\underline{L}) $$

for $X,Y\in \Gamma (T\mathcal {N})$, where $\overline{\nabla }$ denotes the Levi-Civita connection of $(\overline{M},\overline{g})$.

Let $(\Sigma , \gamma )$ be an orientable spacelike codimension-2 surface in $(\overline{M},\overline{g})$. Recall that the *vector-valued second fundamental form* of Σ in (*M*, *g*) is defined as

$$ \vec {\operatorname {II}}(V,W)=\left( \overline{\nabla }_VW\right) ^\perp $$

for all tangent vector fields $V, W\in \Gamma (T\Sigma )$. Further, the *codimension-2 mean curvature vector* $\vec {\mathcal {H}}$ of Σ is given by the trace of $\vec {\operatorname {II}}$ with respect to γ, i.e., $\vec {\mathcal {H}}=\text {tr}_\gamma \vec {\operatorname {II}}$. Additionally, we define the *spacetime mean curvature* $\mathcal {H}^2$ as the Lorentzian length of $\vec {\mathcal {H}}$, i.e.,

$$ \mathcal {H}^2=\overline{g}(\vec {\mathcal {H}},\vec {\mathcal {H}}). $$

If $\vec {\mathcal {H}}$ is spacelike, this agrees with the notion of spacetime mean curvature by Cederbaum–Sakovich [18] upon taking a square root. However, as $\vec {\mathcal {H}}$ will in general have no fixed causal character, $\mathcal {H}^2$ can be at least locally negative. Indeed, *trapped surfaces*, where $\vec {\mathcal {H}}$ is timelike everywhere along Σ, arise naturally in the context of general relativity. Additionally, we call Σ a surface of constant spacetime mean curvature (STCMC surface) if $\mathcal {H}^2$ is constant along Σ; see [18]. In terms of the spacetime mean curvature, the Hawking energy $m_H(\Sigma )$ of Σ is given by

$$ m_H(\Sigma )=\sqrt{\frac{\left| \Sigma \right| }{16\pi }}\left( 1-\frac{1}{16\pi }\int _\Sigma \mathcal {H}^2\,\textrm{d}\mu \right) . $$

Let $\{\underline{L},L\}$ be a null frame of $\Gamma (T^\perp \Sigma )$ with $g(\underline{L},L)=2$.^3^ Then, $\vec {\operatorname {II}}$ and $\vec {\mathcal {H}}$ admit the decomposition

1 $$\begin{aligned} \begin{aligned} \vec {\operatorname {II}}&=-\frac{1}{2}\chi \underline{L}-\frac{1}{2}\underline{\chi }L,\\ \vec {\mathcal {H}}&=-\frac{1}{2}\theta \underline{L}-\frac{1}{2}\underline{\theta }L, \end{aligned} \end{aligned}$$

where the null second fundamental forms $\underline{\chi }$ and χ with respect to $\underline{L}$ and *L*, respectively, are defined as

$$\begin{aligned} \underline{\chi }(V,W):=-\overline{g}\left( \overline{\nabla }_VW,\underline{L} \right)&\,&{\chi }(V,W)&:=-\overline{g}\left( \overline{\nabla }_VW,L \right) , \end{aligned}$$

for tangent vector fields $V,W\in \Gamma (T\Sigma )$, and the null expansions $\underline{\theta }$ and θ with respect to $\underline{L}$ and *L*, respectively, as

$$\begin{aligned} \underline{\theta }:= \operatorname {tr}_\gamma \underline{\chi },&\,&{\theta }:= \operatorname {tr}_\gamma {\chi }. \end{aligned}$$

Under the conventions taken here, the (twice contracted) Gauss equation becomes

2 $$\begin{aligned} \overline{\textrm{R}}-2\overline{\textrm{Ric}}(\underline{L},L)+\frac{1}{2}\overline{\operatorname {Rm}}(\underline{L},L,L,\underline{L})=\textrm{R}-\mathcal {H}^2+\vert \vec {\operatorname {II}}\vert ^2, \end{aligned}$$

where $\textrm{R}$ denotes the scalar curvature of Σ and (1) implies that

3 $$\begin{aligned} \vert \vec {\operatorname {II}}\vert ^2=\langle \underline{\chi },\chi \rangle ,\text { and }\mathcal {H}^2=\underline{\theta }\theta . \end{aligned}$$

See, for example, [3, Equation (9)], and see [14, Proposition 4.24] for a proof using the same conventions. We further recall that the connection-1 form ζ of Σ (with respect to $\{\underline{L},L\}$) is defined as

$$ \zeta (V):=\frac{1}{2}g\left( \overline{\nabla }_V\underline{L},L \right) . $$

In the following, we will always assume that Σ is a spacelike cross section of a given null hypersurface $\mathcal {N}$, i.e., Σ is a spacelike codimension-2 surface of $(\overline{M},\overline{g})$ such that $\Sigma \subseteq \mathcal {N}$ and all integral curves of the null generator $\underline{L}$ intersect Σ exactly once. For a spacelike cross section Σ of $\mathcal {N}$, we shall always take the normal null vector field $\underline{L}\in \Gamma (T^\perp \Sigma )$ to be the given choice of null generator $\underline{L}$. We note that a null generator $\underline{L}$ of $\mathcal {N}$ is orthogonal to all spacelike cross sections by definition. Additionally, we note that we may use $\underline{\chi }$ in this case both for the spacelike cross section Σ and the null hypersurface $\mathcal {N}$ without ambiguity as

$$ \underline{\chi }_\Sigma =\underline{\chi }_{\mathcal {N}}(\,\cdot \,\vert _{T\Sigma },\,\cdot \,\vert _{T\Sigma }). $$

In particular, we may extend $\underline{\theta }$ to a smooth function on $\mathcal {N}$ via a foliation of spacelike cross sections. In fact, it is well known that $\underline{\theta }$ extends to a well-defined function on $\mathcal {N}$ that is independent of the choice of foliation. See, e.g., [14, Section 4.7 and 4.8] for more details regarding this standard fact, where the same notation and conventions are used. That is to say, the expansion $\underline{\theta }_\Sigma$ for any spacelike cross section of $\mathcal {N}$ is the restriction of $\underline{\theta }_\mathcal {N}$ to Σ, and again we simply use $\underline{\theta }$ by slight abuse of notation without ambiguity. In particular, $\underline{\theta }$ is only pointwise dependent on the spacelike cross section. We adopt the following definition by Roesch [10, Definition 2.1]:

### Definition 1

We say $\mathcal {N}$ is a *null cone* if there exists a null generator $\underline{L}$ such that $\underline{\theta }>0$ on $\mathcal {N}$.

Examples of null cones are the (standard) lightcones in the Minkowski and Schwarzschild spacetime; see Sect. 4. Note that for null hypersurfaces modeling black hole and cosmological horizons one has $\underline{\theta }=0$ throughout $\mathcal {N}$. In this sense, it is a natural restriction to assume that $\mathcal {N}$ is a null cone when one does not aim to model horizons. However, in general a null hypersurface is neither a null cone nor a horizon.

Let Σ be a spacelike cross section with $\underline{\theta }>0$. Then, the *torsion* τ and *scalar second fundamental form* *A* are defined as

$$\begin{aligned} \tau&:=\zeta -\,\textrm{d}\ln \underline{\theta },\\ A&:=\underline{\theta }\chi , \end{aligned}$$

see [19, Section 1] and [20, Section 3]. In particular, these objects are always well defined for a spacelike cross section of a null cone. Moreover, it is easy to check that τ, *A*, $\mathcal {H}^2=\text {tr}A$, $\underline{\theta }^{-1}\underline{L}$, $\underline{\theta }^{-1}\underline{\chi }$ are well defined and independent of the choice of null generator $\underline{L}$. That is, these well-defined objects on a null cone encode the geometry of Σ and $\mathcal {N}$ independent of the gauge choice.

## The stability operator

Let $\mathcal {N}$ be a null cone, Σ a spacelike cross section of $\mathcal {N}$, and let $x:(-\varepsilon ,\varepsilon )\times \Sigma \rightarrow \mathcal {N}$ be a (local) smooth variation of Σ along $\mathcal {N}$ with

$$ \frac{\,\textrm{d}}{\,\textrm{d}t}x\big |_{t=0}=\varphi \underline{L}. $$

By the well-known Raychaudhuri optical equations, see, e.g., [14, Proposition 4.27] for a proof using the same conventions, we note that

$$\begin{aligned} \frac{\,\textrm{d}}{\,\textrm{d}t}\left| \Sigma \right| \big |_{t=0}=\int _{\Sigma }\underline{\theta }\varphi \,\textrm{d}\mu ,\\ \frac{\,\textrm{d}}{\,\textrm{d}t}\mathcal {H}^2\big |_{t=0}=Q(\varphi ), \end{aligned}$$

where *Q* is a second-order linear operator which depends on the choice of null generator $\underline{L}$. In [11, Section 3.2], Kröncke and the author observed that

$$ Q(\varphi )=J(\underline{\theta }\varphi ), $$

where the second-order elliptic operator *J* is of divergence type and given by4where  denotes the trace-free part of a (0, 2)-tensor *T* on $(\Sigma ,\gamma )$.

We call *J* the Jacobi operator of Σ and note that it is independent of the choice of null generator $\underline{L}$. From this perspective, it does seem convenient to rephrase the variation of a smooth family of spacelike cross sections $x:(-\varepsilon ,\varepsilon )\times \Sigma \rightarrow \mathcal {N}$ along a null cone as

$$ \frac{\,\textrm{d}}{\,\textrm{d}t}x\big |_{t=0}=f\underline{\theta }^{-1}\underline{L}, $$

such that

$$\begin{aligned} \frac{\,\textrm{d}}{\,\textrm{d}t}\left| \Sigma \right| \big |_{t=0}=\int _{\Sigma }f\,\textrm{d}\mu ,\\ \frac{\,\textrm{d}}{\,\textrm{d}t}\mathcal {H}^2\big |_{t=0}=J(f). \end{aligned}$$

Exactly as in [11, Definition 3.8], we now consider a notion of stability (under area-preserving variations) along null cones that can be understood as an analogue to the stability of CMC surfaces in the Riemannian setting (although we do not assume the STCMC condition here).

### Definition 2

Let $\mathcal {N}$ be a null cone, Σ a spacelike cross section of $\mathcal {N}$. We say Σ is stable (under area-preserving variations) if there exists a nonnegative constant c≥0 such that

$$ \int _\Sigma fJ(f)\,\textrm{d}\mu \ge c\int _\Sigma f^2\,\textrm{d}\mu $$

for all $f\in W^{1,2}(\Sigma )$ such that $\int _\Sigma f\,\textrm{d}\mu =0$. We say Σ is strictly stable (under area-preserving variations), if Σ is stable with c>0.

Here, $W^{1,2}(\Sigma )$ denotes a weighted Sobolev norm as defined in [11, Section 2.2], but one may also consider the definition with unweighted norms.

### Remark 1

Observe that Eq. (3) and the Gauss Eq. (2) yield thatwhere we recall that $\textrm{R}$ denotes the scalar curvature of Σ and ${G}=\overline{\textrm{Ric}}-\frac{1}{2}\overline{R}\overline{g}$ denotes the Einstein tensor of $(\overline{M},\overline{g})$. Asintegration by parts yields that

$$\begin{aligned}&\int _\Sigma fJ(f)\,\textrm{d}\mu =\,\int _\Sigma 2\left| \nabla f\right| ^2\,\textrm{d}\mu -\int _\Sigma f^2\\  &\quad \left[ \mathcal {H}^2\left( 1+ \underline{\theta }^{-2}\left( \vert \underline{\chi }\vert ^2+\overline{\textrm{Ric}} (\underline{L},\underline{L})\right) \right) -\textrm{R}-{G} (\underline{L},L)+2\left| \tau \right| ^2\right] \,\textrm{d}\mu . \end{aligned}$$

### Remark 2

In their previous work, Kröncke and the author connect (strict) stability in the sense of Definition 2 to a (positive) lower bound on the first eigenvalue of *J* via a Rayleigh quotient, see [11, Remark 3.9]. While this is true for the surfaces under consideration in [11], the connection is more subtle than initially stated in [11] and in fact false in the general case. To clarify, let us expand on [11, Remark 3.9] here: Let us first comment that in general *J* is not self-adjoint, so eigenvalues of *J* have to be defined similar as in [2] in analogy to the notion of MOTS stability. To circumvent this subtlety, one may consider the self-adjoint operator ${\widetilde{J}(f):=J(f)+4\tau (\nabla f)+2\operatorname {div}\tau f}$ as

$$ \int _{\Sigma } fJ(f)\,\textrm{d}\mu =\int _{\Sigma }f\widetilde{J}(f)\,\textrm{d}\mu . $$

Note that $\widetilde{J}=\frac{1}{2}(J+J^*)$, where $J^*$ denotes the adjoint of *J*, and that $\widetilde{J}=J=J^*$ if $\tau \equiv 0$, which is, for example, always satisfied in Class $\mathcal {S}$ (see Sect. 4). Even though $\widetilde{J}$ now has a discrete, real spectrum

$$ \lambda _0\le \lambda _1\le \dotsc , $$

it is in general not clear whether $\lambda _0<\lambda _1$ or if constant functions are eigenfunctions with respect to $\lambda _0$. Hence, stability in the sense of Definition 2 does in general not infer any information on $\lambda _1$. However, all of the above is true in the case of coordinate spheres $\mathbb {S}^2_r$ in the Schwarzschild lightcone (of positive mass m>0) where constant functions are eigenfunctions with respect to the eigenvalue $\lambda _0$ and

$$ \lambda _0=-\frac{4}{r^2}+\frac{12\,m}{r^3}<\frac{12\,m}{r^3}=\lambda _1. $$

Here, we recall that in [11], the null hypersurface under consideration is assumed to be close to the Schwarzschild lightcone in a strong sense, see [11, Definition 2.10], and spacelike cross sections are always assumed to be contained in a very restrictive a-priori class of surface $B_\sigma (B_1,B_2,B_3)$, see [11, Definition 3.10], both of which enforce closeness to coordinate spheres in the Schwarzschild lightcone. Hence, while not immediately implied by the strict stability, one still finds that in this case the eigenfunctions with respect to $\lambda _0$ are close to constants and that

$$ \lambda _0\le -\frac{4}{\sigma ^2}+\frac{Cm}{\sigma ^3}<0<\frac{\varepsilon m}{\sigma ^3}\le \lambda _1 $$

for suitable uniform constants ε<12<C provided σ is sufficiently large.

### Remark 3

While the results below and the applications in [11] indicate that stability in the sense of Definition 2 appears to be the direct null analogue for CMC stability in the case of a null cone, we note that there are many notions of stability and generalizations of CMC stability in the context of General Relativity. Here, we also want to mention the related notions of ${\textbf {H}}$*-stability* by Alaee–Lesourd–Yau [1] and *variational stability* by Peñuela Diaz [9]. In all cases, the stability operator is derived from the variation of the spacetime mean curvature. However, unlike here, where the variation is considered only along the given null cone, stability in [1, 9] is formulated as a codimension-2 property directly in the ambient spacetime with respect to a general normal direction in [1] and with respect to the direction of the mean curvature vector $\vec {\mathcal {H}}$ in [9], respectively. In fact, stability in the sense of Definition 2 arises as a special case from the notion of ${\textbf {H}}$-stability (under a volume-preserving condition) in [1] when taken in the null direction $\underline{\theta }^{-1}\underline{L}$.

In analogy to the work of Christodoulou–Yau [12], we derive a nonnegative lower bound for the Hawking energy of stable spacelike cross sections. Similar bounds for stable surfaces are derived in [1, 9], and we will utilize the same set of test functions here. Since stability in the sense of Definition 2 is a special case of ${\textbf {H}}$-stability (under a volume-preserving condition) as in [1], the nonnegativity of the Hawking energy directly follows from [1, Theorem 3.3]. Here, we present a slightly refined estimate and include a precise statement and proof for the convenience of the reader.

### Proposition 1

Let $\mathcal {N}$ be a null cone with spherical cross sections in an ambient 4-dimensional spacetime $(\overline{M},\overline{g})$ that satisfies the DEC, and let Σ be a spacelike cross section with $\mathcal {H}^2\ge 0$. If Σ is stable (with respect to area-preserving variations) with constant c≥0, then

$$ m_{H}(\Sigma )\ge \frac{2}{3}c\left( \frac{\left| \Sigma \right| }{16\pi }\right) ^{\frac{3}{2}}. $$

### Remark 4

If c>0, we note that the DEC may be replaced by a suitable smallness assumption on $\overline{\textrm{Ric}}$. Indeed, for the asymptotic foliation by stable STCMC surfaces constructed by Kröncke and the author in [11], the constant *c* as established in [11, Proposition 3.10] and the decay assumptions on the curvature in [11, Definition 2.10] are such that one can still infer the positivity of the Hawking energy along the leaves of the foliation. Note that the positivity of the Hawking mass along the leaves of the foliation has independently been established by Peñuela Diaz in [9] where he shows that the leaves of the foliation are also variationally stable, see [9, Theorem 5.4], in the sense of [9, Definition 3.2].

### Proof

Note that as $-\underline{L}$ and *L* are either both past- or future pointing, we have

$$ \overline{\textrm{Ric}}(\underline{L},\underline{L})\ge 0,\text { and }-{G}(\underline{L},L)={G}(-\underline{L},L)\ge 0 $$

by the dominant energy condition. Hence, by Remark 1, we find that

$$ \int _\Sigma \left[ c+\frac{3}{2}\mathcal {H}^2\right] f^2\,\textrm{d}\mu \le \int _\Sigma 2\left| \nabla f\right| ^2+\textrm{R}f^2\,\textrm{d}\mu . $$

for any $f\in W^{1,2}(\Sigma )$ with $\int _\Sigma f\,\textrm{d}\mu =0$. Since Σ is a topological 2-sphere, there exists functions $\widetilde{f}_i$, i=1,2,3, such that

$$ \left[ \int _\Sigma \widetilde{f}_i\,\textrm{d}\mu =0,\,\sum \limits _{i=1}^3\widetilde{f}_i^2=1,\,\sum \limits _{i=1}^3\int _\Sigma \left| \nabla \widetilde{f}_i\right| ^2\,\textrm{d}\mu =8\pi \right] , $$

arising from a conformal mapping by Hersch’s lemma [21]. Hence, using these as test functions and summing over *i*, the above integral estimate and the Gauss–Bonnet theorem imply that

$$ \int _\Sigma \mathcal {H}^2\,\textrm{d}\mu \le 16\pi -\frac{2}{3}c\left| \Sigma \right| . $$

We conclude that

$$\begin{aligned} m_H(\Sigma )=\sqrt{\frac{\left| \Sigma \right| }{16\pi }}\left( 1-\frac{1}{16\pi }\int _\Sigma \mathcal {H}^2\,\textrm{d}\mu \right) \ge \sqrt{\frac{\left| \Sigma \right| }{16\pi }}\left( 1-1+\frac{c}{24\pi }\left| \Sigma \right| \right) =\frac{2}{3}c\left( \frac{\left| \Sigma \right| }{16\pi }\right) ^{\frac{3}{2}}.\\ \end{aligned}$$

□

We can derive the following rigidity statement:

### Proposition 2

Let $\mathcal {N}$ be a null cone with spherical cross sections in an ambient 4-dimensional spacetime $(\overline{M},\overline{g})$ that satisfies the DEC, and let Σ be a stable spacelike cross section with $\mathcal {H}^2\ge 0$. If

$$ m_{H}(\Sigma )=0 $$

and $\overline{\textrm{Ric}}(\underline{L},L)-\frac{1}{2}\operatorname {Rm}(\underline{L},L,L,\underline{L})$ does not change sign along Σ, then Σ embeds isometrically as a stable spacelike cross section into the standard lightcone of the Minkowski spacetime $(\mathbb {R}^{1,3},\eta )$ such that

$$ \eta (\vec {\mathcal {H}}_{Mink},\vec {\mathcal {H}}_{Mink})=\mathcal {H}^2=g(\vec {\mathcal {H}},\vec {\mathcal {H}}), $$

where $\vec {\mathcal {H}}_{Mink}$ denotes the mean curvature vector of the embedding of Σ into $(\mathbb {R}^{1,3},\eta )$.

### Remark 5

We note that the assumption on the sign of $\overline{\textrm{Ric}}(\underline{L},L)-\frac{1}{2}\operatorname {Rm}(\underline{L},L,L,\underline{L})$ is closely related to a similar assumption in the recent rigidity result by Peñuela Diaz in [9, Theorem 4.1]. While we essentially show that $\mathcal {N}$ agrees with the Minkowski lightcone along Σ, the characterization by Peñuela Diaz derived from variational stability in [9] goes further. That is, Peñuela Diaz shows that Σ is a round sphere and that any spacelike hypersurface Ω in $\overline{M}$ with $\partial \Omega =\Sigma$ embeds isometrically into the Minkowski spacetime. By analogy, it is thus reasonable to expect that the assumptions in Proposition 2 in fact imply that $\mathcal {N}$ is (a part of) the standard Minkowski lightcone and that Σ is a round sphere. Since we show that Σ embeds as a stable spacelike cross section, Corollary 4 indeed implies that Σ is an STCMC surface, i.e., Σ is a round sphere and $\mathcal {H}^2$ is a strictly positive constant.

### Proof

By assumption, Σ is a topological 2-sphere. Hence, the uniformization theorem implies that its induced metric γ is conformally round, i.e., $\gamma =\omega ^2\widehat{\gamma }$ for some positive function $\omega :\mathbb {S}^2\rightarrow (0,\infty )$ where $\widehat{\gamma }$ denotes the standard round metric. It is a well-known fact that Σ then embeds isometrically into the (future-directed and past-directed) standard lightcone in the Minkowski spacetime $(\mathbb {R}^{1,3},\eta )$ via the identification $\Sigma =\{\pm t=r=\omega \}$. See, for example, [20]. See also Sect. 4. By the Gauss equation in the Minkowski spacetime, see, e.g., [20, Remark 1], we have

$$ \eta (\vec {\mathcal {H}}_{Mink},\vec {\mathcal {H}}_{Mink})=2\textrm{R}, $$

so it remains to show that $\mathcal {H}^2=2\textrm{R}$ and that the embedding of Σ into the Minkowski lightcone is stable. By the Gauss equation in $(\overline{M},\overline{g})$ as stated in Remark 1, we haveNote that by Proposition 1, $m_H(\Sigma )=0$ shows that stability necessarily holds with constant c=0. Then, using the same test functions as above a closer examination of the integration by parts in Remark 1 yields the following chain of inequalities

$$\begin{aligned} \frac{3}{2}\int _\Sigma \mathcal {H}^2\,\textrm{d}\mu \!\!\le \!\!\int _\Sigma \mathcal {H}^2\left[ 1+\underline{\theta }^{-2}\left( \vert \underline{\chi }\vert ^2+\overline{\textrm{Ric}}(\underline{L},\underline{L})\right) \right] -{G}(\underline{L},L)+2\left| \tau \right| ^2\,\textrm{d}\mu \le 24\pi . \end{aligned}$$

By assumption, equality now holds everywhere. In particular, we find that , $\vert \tau \vert =0$ and ${G}(\underline{L},L)=0$, $\overline{\textrm{Ric}}(\underline{L},\underline{L})=0$ along Σ as we assume the DEC. The above Gauss equation hence simplifies to

$$ \mathcal {H}^2-2\textrm{R}=2\overline{\textrm{Ric}}(\underline{L},L)-\overline{\operatorname {Rm}}(\underline{L},L,L,\underline{L}). $$

Further, by assumption and the Gauss–Bonnet theorem integration now yields that

$$ \int _\Sigma 2\overline{\textrm{Ric}}(\underline{L},L)-\overline{\operatorname {Rm}}(\underline{L},L,L,\underline{L})\,\textrm{d}\mu =0. $$

However, $2\overline{\textrm{Ric}}(\underline{L},L)-\overline{\operatorname {Rm}}(\underline{L},L,L,\underline{L})$ has a fixed sign by assumption, so it in fact must vanish identically. Hence, $\mathcal {H}^2=2\textrm{R}$. We will now argue that the stability operator *J* similarly simplifies to its Minkowski counterpart. Since Σ is stable with respect to *J* by assumption, this readily implies that Σ is stable in the Minkowski lightcone. By the above arguments, it is immediate that *J* as given in (4) simplifies to

$$ J(f)=-2\Delta f-\mathcal {H}^2f=-2\Delta f-2\textrm{R}f. $$

One can directly verify, see Sect. 4, that this is the form of the stability operator for cross sections of the Minkowski lightcone. □

## The stability operator in class $\mathcal {S}$

We now consider a class $\mathcal {S}$ of spacetimes of the form $\overline{M}=\mathbb {R}\times I\times \mathbb {S}^2$ and

$$ \overline{g}=-h(r)\,\textrm{d}t^2+\frac{1}{h(r)}\,\textrm{d}r^2+r^2\widehat{\gamma }, $$

where $h:(0,\infty )\rightarrow \mathbb {R}$ is a smooth function, *I* is an open interval on which $\left| h\right|$ is strictly positive, and $\widehat{\gamma }$ denotes the standard round metric on $\mathbb {S}^2$. Spacetimes of class $\mathcal {S}$ have been widely studied and although they are highly rigid, they contain many physically relevant toy models such as the Schwarzschild spacetime corresponding to $h(r)=1-\frac{2m}{r}$. See, for example, [22] and the references given therein. We observe that any zeros $r_i$ of *h* correspond to a Killing horizon in a suitable spacetime extension. The null cones we consider here are the principle null hypersurfaces generated by null geodesics (not tangential to a Killing horizon) starting orthogonally from a coordinate sphere.

For the sake of brevity, we will collect the relevant properties of a principal null hypersurface $\mathcal {N}$ as above without proof. While most of these identities are well known and can be derived in the original coordinate chart, it is particularly convenient to perform these computations in a suitable double null coordinate system (*u*, *v*) in which these principal null hypersurfaces are given as level sets of the coordinate functions *u* and *v*. Besides the Minkowski spacetime, where one simply takes v=r+t, u=r-t, such double null coordinates also arise from a spacetime extension in spirit of the Kruskal–Szekeres extension. See, e.g., [22–24]. While this specific spacetime extension requires that there exists a non-degenerate Killing horizon, here $h'(r_i)\not =0$, this is a natural physical assumption that is satisfied by most toy models. In such coordinates, all relevant properties of $\mathcal {N}$ have been derived in [25, Section 3]. We leave it as an exercise to the reader to derive these identities in the original (*t*, *r*) coordinates and always assume implicitly that double null coordinates as above exist for simplicity. In particular, we refer the reader to [25, Section 3] for details and computations and only summarize the relevant properties of $\mathcal {N}$ below:

There exists a choice of geodesic null generator $\underline{L}$ of $\mathcal {N}$ such that $\underline{L}(r)=1$. In particular, since any spacelike cross section Σ can be realized as a graph with respect to a choice of background foliation, this specific choice of $\underline{L}$ shows that one can realize any spacelike cross section Σ via ${\Sigma =\{r=\omega \}\cap \mathcal {N}}$ with $\omega :\mathbb {S}^2\rightarrow (0,\infty )$. Note that ω is simultaneously the conformal factor of the induced metric $\gamma =\omega ^2\widehat{\gamma }$. Moreover, for any spacelike cross section Σ of $\mathcal {N}$ we have

$$\begin{aligned} \underline{\chi }&=\omega \widehat{\gamma },\\ \underline{\theta }&=\frac{2}{\omega }>0,\\ \tau&= 0,\\ \mathcal {H}^2&=2\textrm{R}-\frac{4(1-h(\omega ))}{\omega ^2}. \end{aligned}$$

While the identity for τ is not given explicitly in [25, Section 3], one can directly verify from the well-known formula for ζ with respect to a given background foliation, see, e.g., [14, Proposition 4.22], and the identities in [25, Section 3] that τ vanishes identically for any spacelike cross section. Combining the above identities with the relevant curvature quantities, which one can derive, for example, from [22, Proposition B.1], we leave it as an exercise to the reader to verify that the Jacobi operator *J* in this setting becomes

$$ J(f)=-2\Delta f-\left( \mathcal {H}^2-\frac{2}{\omega }h'(\omega )\right) f $$

by direct computation.

Using Hersch’s lemma [21] as before as well as the above identity for *J* and $\mathcal {H}^2$ as above, stability in the sense of Definition 2 (with c=0) now implies

$$ 0\le \int _\Sigma \frac{4(1-h(\omega ))}{\omega ^2}+\frac{2\,h'(\omega )}{\omega }\,\textrm{d}\mu , $$

i.e., any stable spacelike cross section Σ must lie in a region of $\mathcal {N}$ where the above function with respect to *h* integrates to a nonnegative number. While this rules out stable cross sections in the Schwarzschild lightcone of negative mass m<0, the above identity trivially satisfies in the Minkowski lightcone and the Schwarzschild lightcone of positive mass m>0. That is, there is a plethora of meaningful choices for *h* for which the usual trick via Hersch’s lemma does not yield any geometric information on stable spacelike cross sections at all.

We recall that the conformal mapping chosen in Hersch’s lemma can also be motivated from a notion of conformal center of mass for topological 2-spheres introduced by Onofri [26]. Essentially equivalent to the choice of conformal mapping in [21], Onofri [26] showed that one can always choose a suitable conformal diffeomorphism on $\mathbb {S}^2$, i.e., a suitable Möbius transformation, such that there exists a conformal factor *w* satisfying

$$ \int _{\mathbb {S}^2}w^2f_i\,\textrm{d}\widehat{\mu }=0 $$

for all i=1,2,3 where the $f_i$ denote (a choice of) first spherical harmonics and $\,\textrm{d}\widehat{\mu }$ is the area element of the standard round sphere $\widehat{\gamma }$. Indeed, the well-known formulas

$$ \sum \limits _{i=1}^3f_i^2=1,\,\sum \limits _{i=1}^r\vert \widehat{\nabla }f_i\vert ^2_{\widehat{\gamma }}=2 $$

directly imply the properties of the functions in Hersch’s lemma. Since any two choices of a round metric on $\mathbb {S}^2$ (with scalar curvature 1) are related by such a Möbius transformation, the above can also be understood as a choice of round metric on $\mathbb {S}^2$.

In a similar spirit, the author showed in [13] that one can always choose a Möbius transformation, i.e., make a choice of round metric, such that the conformal factor *w* with respect to this choice satisfies the balancing condition

5 $$\begin{aligned} \int _{\mathbb {S}^2}w^3f_i\,\textrm{d}\widehat{\mu }=0 \end{aligned}$$

for (a choice of) first spherical harmonics $f_i$. The balancing condition (5) is derived from a timelike 4-vector defined for spacelike cross sections of the Minkowski lightcone. Projecting this Minkowskian 4-vector to the hyperboloid (of radius 1), one can uniquely associate a vector $\vec {a}\in \mathbb {R}^3$ to a given conformally round metric $\omega ^2\widehat{\gamma }$. See also [27]. As this Minkowskian 4-vector is shown to transform equivariantly under Lorentz transformations in $\operatorname {SO}^+(1,3)$, we note $\vec {a}$ depends on the choice of round metric in a natural way. Moreover, this Lorentz equivariance further implies that the balancing condition (5) is always satisfied by

6 $$\begin{aligned} w(\vec {x})=\frac{\omega \circ \Phi _{-\vec {a}}(\vec {x})}{\sqrt{1+\left| \vec {a}\right| ^2}+\vec {a}\cdot \vec {x}}, \end{aligned}$$

where $\vec {x}$ is understood as a point on $\mathbb {S}^2$ via the canonical embedding into $\mathbb {R}^3$. In particular, the Euclidean inner product $\vec {a}\cdot \vec {x}$ is understood as a sum of first spherical harmonics, i.e., $\vec {a}\cdot \vec {x}=\vec {a}^if_i$ as a function on $\mathbb {S}^2$. Here, $\Phi _{-\vec {a}}$ is the Möbius transformation uniquely determined by the Lorentz transformation in direction $-\vec {a}$ via the isomorphism of $\operatorname {SO}^+(1,3)$ and the Möbius group. Hence, $\Phi _{-\vec {a}}$ is precisely the conformal diffeomorphism that relates the two choices of round metric such that $\gamma =\omega ^2\widehat{\gamma }$ becomes $\gamma =w^2\widehat{\gamma }$, where we use $\widehat{\gamma }$ for both metrics by slight abuse of notation as they are related via this coordinate transformation. We refer the interested reader to [13, Section 3] for more details. Here, we merely stress again that while the Minkowskian 4-vector is understood from an extrinsic perspective in [13], the balancing condition (5) can also be derived from a purely intrinsic perspective for a conformally round metric. Thus, although Lorentz transformations are in general not isometries of the ambient spacetime of class $\mathcal {S}$, for a given spacelike cross section Σ in $\mathcal {N}$ as above one can always make a choice of round metric $\widehat{\gamma }$ such that the induced metric satisfies $\gamma =w^2\widehat{\gamma }$ with *w* satisfying (5). While this *balanced* conformal factor no longer corresponds to the graph function ω, we note that (6) yields that

7 $$\begin{aligned} \omega (\vec {x})=\frac{w\circ \Phi _{\vec {a}}(\vec {x})}{\sqrt{1+\left| \vec {a}\right| ^2}-\vec {a}\cdot \vec {x}}. \end{aligned}$$

As a consequence, the balancing condition (5) yields new test functions for stable surfaces:

### Proposition 3

Let $\mathcal {N}$ be a principal null hypersurface in a spacetime of class $\mathcal {S}$. Let Σ be a stable spacelike cross section in $\mathcal {N}$ with graph function ω. Then,

$$ \int _{\mathbb {S}^2}\vert \widehat{\nabla }w\vert _{\widehat{\gamma }}^2\,\textrm{d}\widehat{\mu }\le \int _{\mathbb {S}^2}\left( \frac{(1-h(\omega ))}{\omega ^2}+\frac{h'(\omega )}{2\omega }\right) w^4\,\textrm{d}\widehat{\mu } $$

for any conformal factor *w* satisfying (5).

### Remark 6

Since the underlying Minkowskian center transforms equivariantly under Lorentz transformations, we note that any two conformal factors $w_1$, $w_2$ satisfying (5) are related by an isometry on $\mathbb {S}^2$, i.e., a rotation.

### Proof

Using the explicit formulas for $\mathcal {H}^2$ and *J* as above, a spacelike cross section Σ is stable in $\mathcal {N}$ if and only if

$$ \int _\Sigma \textrm{R}f^2\,\textrm{d}\mu \le \int _\Sigma \left| \nabla f\right| ^2+\left( \frac{2(1-h(\omega ))}{\omega ^2}+\frac{h'(\omega )}{\omega }\right) f^2\,\textrm{d}\mu $$

for all $f\in W^{1,2}(\Sigma )$ with $\int _\Sigma f\,\textrm{d}\mu =0$. Using a Möbius transformation as above, we may shift to coordinates such that $\gamma =w^2\widehat{\gamma }$ where the conformal factor *w* satisfies the balancing condition (5) and where the graph function ω on $\Sigma \cong \mathbb {S}^2$ is now expressed via (7). In particular,

$$ \int _{\Sigma }wf_i\,\textrm{d}\mu =0 $$

for first spherical harmonics $f_i$, i=1,2,3. We recall that

$$ \sum \limits _{i=1}^3f_i^2=1,\text { and }\sum \limits _{i=1}^3\vert \widehat{\nabla }f_i\vert _{\widehat{\gamma }}^2=2, $$

and we moreover observe that

$$ \sum \limits _{i=1}^3f_i\widehat{\gamma }(\widehat{\nabla } f_i,\widehat{\nabla }w)=0 $$

for any $C^1$ function *w*.^4^ Observe further that

$$ \int _\Sigma \left| \nabla f\right| ^2\,\textrm{d}\mu =\int _{\mathbb {S}^2}\vert \widehat{\nabla }f\vert _{\widehat{\gamma }}^2\,\textrm{d}\widehat{\mu } $$

for any $C^1$ function *f*. Hence, using $wf_i$ as test functions for the stability and summing over *i*, the above identities and the conformal change of the area element yield that

$$ \int _\Sigma \textrm{R}w^2\,\textrm{d}\mu \le \int _\Sigma 2+\left| \nabla w\right| ^2+\left( \frac{2(1-h(\omega ))}{\omega ^2}+\frac{h'(\omega )}{\omega }\right) w^2\,\textrm{d}\mu . $$

As $\gamma =w^2\widehat{\gamma }$, the well-known formula for the scalar curvature yields

$$ w^2\textrm{R}=2+2\left| \nabla w\right| ^2-w\Delta w. $$

Hence, integration by parts and simplifying on both sides yields

$$ 2\int _\Sigma \left| \nabla w\right| ^2\,\textrm{d}\mu \le \int _\Sigma \left( \frac{2(1-h(\omega ))}{\omega ^2}+\frac{h'(\omega )}{\omega }\right) w^2\,\textrm{d}\mu . $$

Invoking the conformal change of the gradient and area element once again, the claim follows. □

The main application of the $L^2$-bound in Proposition 3 here is a characterization of stable spacelike cross sections in the Minkowski lightcone.

### Corollary 4

Let $\mathcal {N}$ be the standard lightcone in the Minkowski spacetime $\mathbb {R}^{1,3}$, Σ a stable spacelike cross section in $\mathcal {N}$. Then, Σ is an STCMC surface, i.e., a round sphere.

### Remark 7

Note that all STCMC surfaces in the Minkowski lightcone have been characterized as round spheres by Wang [28]. See also [29] and a recent proof by Palomo–Romero [30]. It is easy to verify that all round spheres are stable and Corollary 4 can be thus understood as an equivalence statement.

### Proof

Since $\mathbb {R}^{1,3}$ corresponds to h=1, we have $\mathcal {H}^2=2\textrm{R}$ and Proposition 3 implies that the balanced conformal factor *w* is constant, i.e., w=ρ>0. Then, (7) implies that

$$ \omega (\vec {x})=\frac{\rho }{\sqrt{1+\left| \vec {a}\right| ^2}-\vec {a}\cdot \vec {x}}. $$

It is a well-known fact that the conformally round metric $\gamma =\omega ^2\widehat{\gamma }$ has constant scalar curvature, see, e.g., [16, 20, 28], i.e., Σ is a round sphere and STCMC surface in $\mathcal {N}$. □

## An explicit variation derived from Lorentz transformations

The previous sections have already highlighted the significance of conformal diffeomorphisms when discussing the stability of topological 2-spheres. Since any topological 2-sphere is conformally round by the uniformization theorem, it admits an isometric embedding into the standard Minkowski lightcone as demonstrated above. In particular, we may utilize Lorentz transformations to generate a canonical variation along the Minkowski lightcone. Again, we emphasize that this extrinsic variation is equivalent to an intrinsic change of coordinates so the following is not limited to the Minkowski lightcone.

Let Σ be a spacelike cross section of the (future-/past-directed) Minkowski lightcone, where we recall that without loss of generality we may always assume that ${\Sigma =\Sigma _\omega =\{\pm t=r=\omega \}}$ and the induced metric γ of Σ satisfies $\gamma =\omega ^2\widehat{\gamma }$ with respect to the graph function ${\omega :\mathbb {S}^2\rightarrow (0,\infty )}$. Let $\vec {a}\in \mathbb {R}^3$ be a unit vector and consider a family of Lorentz transformations $\{\Lambda _{\vec {a},t}\}_{t\in (-1,1)}$ in direction $t\vec {a}$. Since the lightcone itself is invariant under Lorentz transformations in $\operatorname {SO}^+(1,3)$, this yields a smooth family of spacelike cross sections $\Sigma _{\vec {a},t}:=\Lambda _{\vec {a},t}(\Sigma )$. Note that $\Sigma =\Sigma _{\vec {a},0}$ and that $\Sigma _{\vec {a},t}=\Sigma _{\omega _{\vec {a},t}}$ with

$$ \omega _{\vec {a},t}=\frac{\omega \circ \Phi _{\vec {a},t}}{\sqrt{1+t^2}-t\vec {a}\cdot \vec {x}}, $$

where $\Phi _{\vec {a},t}$ is a uniquely determined Möbius transformation on $\mathbb {S}^2$, see, e.g., [13, Section 3]. Although the precise form of the variation in *t*

$$ \frac{\,\textrm{d}}{\,\textrm{d}t}\omega _{\vec {a},t}=\frac{\widehat{\gamma }(\widehat{\nabla }\omega ,\frac{\,\textrm{d}}{\,\textrm{d}t}\Phi _{\vec {a},t})}{\sqrt{1+t^2}-t\vec {a}\cdot \vec {x}}-\frac{\omega _{\vec {a},t}}{\sqrt{1+t^2}-t\vec {a}\cdot \vec {x}}\left( \frac{t}{\sqrt{1+t^2}}-\vec {a}\cdot \vec {x}\right) $$

is not very instructive at first sight, we recall the well-known facts that the area is preserved and the scalar curvature changes via a conformal diffeomorphism, i.e.,

$$\begin{aligned} \left| \Sigma _{\vec {a},t}\right|&=\left| \Sigma \right| ,\\ \textrm{R}_{\vec {a},t}&=\textrm{R}_{\vec {a},0}\circ \Phi _{\vec {a},t}. \end{aligned}$$

Again, see, for example, [13, Section 3]. We now define

$$ f_{\vec {a}}:=\frac{2}{\omega }\frac{\,\textrm{d}}{\,\textrm{d}t}\omega _{\vec {a},t}\vert _{t=0}=2\widehat{\gamma }(\widehat{\nabla }\ln \omega ,\frac{\,\textrm{d}}{\,\textrm{d}t}\Phi _{\vec {a},t}\vert _{t=0})+2 \vec {a}\cdot \vec {x}. $$

Since we have implicitly chosen the null generator $\underline{L}$ such that $\underline{\theta }=\frac{2}{\omega }$, and $\mathcal {H}^2=2\textrm{R}$ by the Gauss equation in Minkowski, the variation formulae at the beginning of Sect. 3 and the above identities for the area and scalar curvature along the variation yield that

8 $$\begin{aligned} \int _\Sigma f_{\vec {a}}\,\textrm{d}\mu =0 \end{aligned}$$

and

9 $$\begin{aligned} -2\Delta f_{\vec {a}}-2\textrm{R}f_{\vec {a}}=J(f_{\vec {a}})=\frac{\,\textrm{d}}{\,\textrm{d}t}\mathcal {H}^2_{\vec {a},t}\vert _{t=0}=2\widehat{\gamma }\left( \widehat{\nabla }R_\Sigma ,\frac{\,\textrm{d}}{\,\textrm{d}t}\Phi _{\vec {a},t}\big |_{t=0}\right) . \end{aligned}$$

We now state the main observation of this section:

### Proposition 5

Let $(\Sigma ,\gamma )$ be a topological 2-sphere. For all $\vec {a}\in \mathbb {S}^2$, we have

$$ \int _{\Sigma }f_{\vec {a}}\,\textrm{d}\mu =\int _{\Sigma }\textrm{R}\cdot f_{\vec {a}}\,\textrm{d}\mu =0. $$

### Proof

We have already seen that $f_{\vec {a}}$ has mean value 0, so it suffices to show that the second integral vanishes as well. As frequently observed above, a Möbius transformation $\Phi _{\vec {a},t}$ is a conformal diffeomorphism on $\mathbb {S}^2$. Hence,

$$ X_{\vec {a}}:=\frac{\,\textrm{d}}{\,\textrm{d}t}\Phi _{\vec {a},t}\big |_{t=0} $$

is a conformal Killing vector field on $\mathbb {S}^2$. By (9),

$$ \int _\Sigma \textrm{R}\cdot f_{\vec {a}}\,\textrm{d}\mu =-\frac{1}{2}\int _\Sigma -2\Delta f_{\vec {a}}-2\textrm{R}f_{\vec {a}}\,\textrm{d}\mu =-\frac{1}{2}\int _{\Sigma }J(f_{\vec {a}})\,\textrm{d}\mu =-\int _\Sigma \widehat{\gamma }(\widehat{\nabla }\textrm{R},X_{\vec {a}})\,\textrm{d}\mu =0, $$

where the last integral vanishes due to the Kazdan–Warner identity. See, for example, Le [31] for a proof utilizing the geometry of the Minkowski lightcone. □

### Remark 8

Proposition 5 can be understood as the null analogue to the integral identity

$$ \int _NH\left\langle \vec {a},\nu \right\rangle \,\textrm{d}\mu _N=0 $$

for a closed surface in $\mathbb {R}^3$ which follows form the divergence theorem. Indeed, one can check that $f_{\vec {a}}=\operatorname {div}_\gamma X_{\vec {a}}$, so Proposition 5 can also be derived directly from the divergence theorem and is essentially an equivalent formulation of the Kazdan–Warner identity. It would be thus interesting to see if one can derive an independent proof of the Kazdan–Warner identity using the Jacobi operator *J*.

While Proposition 5 is the main point of interest in this section, we note that the functions $f_{\vec {a}}$ have mean zero and can thus be used as test functions. For the sake of completeness, we briefly state all relevant pointwise identities for applications in the future:

### Proposition 6

Let $(\Sigma ,\gamma )$ be a conformally round surface with conformal factor ω. Then, there exist functions $f_i$, i=1,2,3, on Σ such that

$$ \int _\Sigma f_i\,\textrm{d}\mu =0 $$

for i=1,2,3 and

$$\begin{aligned} \sum \limits _{i=1}^3f_i^2&=1+\vert \widehat{\nabla }\ln \omega \vert ^2,\\ \sum \limits _{i=1}^3\left| \widehat{\nabla } f_i\right| ^2&=\vert \widehat{\nabla }^2\ln \omega \vert ^2+\vert \widehat{\nabla }\ln \omega \vert ^2+\omega ^2\textrm{R}_\Sigma . \end{aligned}$$

Moreover, the functions $f_i$ span the kernel of $\Delta +\textrm{R}_\Sigma$ if and only if $(\Sigma ,\gamma )$ has constant curvature.

### Proof

The proof is building on the above considerations and purely computational in nature. For the convenience of the reader, we give a brief outline:

Consider $f_i:=\frac{1}{2}f_{\partial _i}$, i=1,2,3. First, we recall the well-known fact that the Lorentz transformation $\Lambda _{\vec {a},t}$ for t≠0 decomposes as

$$ \Lambda _{\vec {a},t}=D_{\vec {a}}\circ \Lambda _t\circ D_{\vec {a}}^{-1}, $$

where $\Lambda _t$ is a special Lorentz boost in direction $t\partial _3$, and $D_{\vec {a}}$ is the uniquely determined rotation mapping $\partial _3$ to $\vec {a}$ (without any rotation perpendicular to $\vec {a}$). See, e.g., [14, Section 4.2]. In particular, the Möbius transformation $\Phi _{\vec {a},t}$ decomposes as

$$ \Phi _{\vec {a},t}=\Phi _{D_{\vec {a}}}\circ \Phi _{t}\circ \Phi _{D^{-1}_{\vec {a}}}, $$

where $\Phi _t$ is the uniquely determined conformal diffeomorphism related to $\Lambda _t$, and $\Phi _{D}$ denotes the unique isometry on $\mathbb {S}^2$ induced via the canonical embedding of $\mathbb {S}^2$ into $\mathbb {R}^3$ and a rotation *D* in $\mathbb {R}^3$. In particular, $\Phi _{D^{-1}_{\vec {a}}}=\Phi ^{-1}_{D_{\vec {a}}}$.

Introducing angular coordinates $(\theta ,\varphi )\in (0,\pi )\times (0,2\pi )$ on $\mathbb {S}^2$, a tedious but straightforward computation yields the following:

**Table:** 

$\vec {a}$	$\vec {a}\cdot \vec {x}$	$X_{\vec {a}}:=\frac{\,\textrm{d}}{\,\textrm{d}t}\Phi _{\vec {a},t}\big |_{t=0}$	$\widehat{\nabla }_\theta X_{\vec {a}}$	$\widehat{\nabla }_\varphi X_{\vec {a}}$
$\partial _1$	$\cos \varphi \sin \theta$	$-\cos \varphi \cos \theta \partial _\theta +\frac{\sin \varphi }{\sin \theta }\partial _\varphi$	$\cos \varphi \sin \theta \partial _\theta$	$\cos \varphi \sin \theta \partial _\varphi$
$\partial _2$	$\sin \varphi \sin \theta$	$-\sin \varphi \cos \theta \partial _\theta -\frac{\cos \varphi }{\sin \theta }\partial _\varphi$	$\sin \theta \sin \varphi \partial _\theta$	$\sin \theta \sin \varphi \partial _\varphi$
$\partial _3$	$\cos \theta$	$\sin \theta \partial _\theta$	$\cos \theta \partial _\theta$	$\cos \theta \partial _\varphi$

Note that in particular, $X_i:=X_{\partial _i}$ are conformal Killing vector fields that span the tangent space at all points on $\mathbb {S}^2$ (covered by the chart of angular coordinates). From this and (9), it is easy to infer that the set of functions $f_i$ span the kernel of $\Delta +\textrm{R}=-\frac{1}{2}J$ if and only if Σ has constant curvature. Finally, the pointwise identities for the set of functions $f_i$ follow from the above identities after another lengthy, but direct computation.

□

## Data Availability

Not applicable.

## Footnotes

1 Note that any two choices of null generators are related by a reparametrization of the integral curves, i.e., by scaling of a nowhere vanishing function.

2 Under suitable additional assumptions, e.g., the existence of a spacelike cross section as defined below, one can in fact globally choose $\underline{L}$ such that its integral curves are null geodesics.

3 Note that according to this convention, $\underline{L}$ and *L* have a different causal character; if one is future-directed, the other is past-directed.

4 An easy way to verify this directly is in angular coordinates $(\theta ,\varphi )\in (0,\pi )\times (0,2\pi )$ where ${\widehat{\gamma }=\,\textrm{d}\theta ^2+\sin ^2\theta ^2\,\textrm{d}\varphi ^2}$, $f_1=\sin \theta \cos \varphi$, $f_2=\sin \theta \sin \varphi$, $f_3=\cos \theta$.
